# The Effects of UV-A Light Provided in Addition to Standard Lighting on Plumage Condition in Laying Hens

**DOI:** 10.3390/ani10061106

**Published:** 2020-06-26

**Authors:** Birgit Spindler, Tammo Weseloh, Christina Eßer, Sarah Katharina Freytag, Lea Klambeck, Nicole Kemper, Robby Andersson

**Affiliations:** 1 Institute for Animal Hygiene, Animal Welfare and Animal Behaviour, University of Veterinary Medicine Hannover, Foundation, D-30173 Hannover, Germany; sarah.freytag@tiho-hannover.de (S.K.F.); nicole.kemper@tiho-hannover.de (N.K.); 2 Animal Husbandry and Poultry Sciences, University of Applied Sciences Osnabrück, D-49090 Osnabrück, Germany; Weseloh-wenkeloh@web.de (T.W.); l.klambeck@hs-osnabrueck.de (L.K.); robby.andersson@uni-osnabrueck.de (R.A.); 3 Gruppenpraxis Meyer-Block, D-49843 Uelsen, Germany; Tina-Arndt@web.de

**Keywords:** behavior, behavioral disorder, feather loss, feather pecking, cannibalism, injury, light spectrum

## Abstract

**Simple Summary:**

Laying hens, like other poultry species, can perceive ultraviolet radiation; nevertheless, standard lighting in poultry houses does not contain UV-A light (UV). However, little is known about the impact of light quality (daylight spectrum by UV-A light illumination) on plumage loss, skin injuries and the production parameters of laying hens. Presuming that offering a daylight spectrum to hens could help to prevent the loss of feathers and skin damage caused by pecking, about 92,000 Lohmann Brown hens with untrimmed beaks were kept on a farm in different barns. In order to measure effects of a daylight spectrum, half of them were kept in poultry housing illuminated with UV-A light provided in addition to standard lighting. The other half was exposed to standard lighting for poultry houses. The results indicate that separately offered UV-A light alone cannot prevent damage to the hens’ integument caused by pecking. In general, complex interactions in conjunction with UV supplementation influenced the birds’ behavior. In order to promote animal health and welfare, further research is necessary to find out more about the appropriate level of UV supplementation and suitable light sources besides optimum light spectrum.

**Abstract:**

Natural light with ultraviolet spectrum (UV) influences the birds´ perception, the reflectivity of their plumage and affects bird behavior. Therefore, in Germany, laying hens kept in barns should be provided with daylight inlets. Nevertheless, lighting in laying hen houses with a UV proportion is not common practice and little is known about the detailed effects of UV-A lighting during the entire rearing and production period. The present on-farm study examines the impact of light quality on plumage loss, skin injuries and production parameters of laying hens. Therefore, about 92,000 Lohmann Brown hens with untrimmed beaks were kept on a farm in two different groups. Half of them were housed in a barn containing 10 pens illuminated by additional UV-A light (simulate “daylight spectrum”). The other half in the second barn were equally grouped, but exposed to standard lighting for poultry houses. Health, production parameters and plumage condition were monitored during rearing and production. The study results reveal that additional UV-A light is associated with the occurrence of plumage damage and cannibalistic injuries during production. In all groups, the plumage condition of the hens was intact when the hens started laying and declined with age. Therefore, complex interactions alongside UV illumination, environmental enrichment, feed and feeding strategies as well as other management factors that possibly affected both feather damage and skin injuries must also be taken into account.

## 1. Introduction

Feather loss and skin damage deriving from feather and skin pecking is a serious welfare problem and may also lead to higher mortality rates in laying hen husbandry. In general, two forms of feather pecking are notable in laying hens [1]. Gentle feather pecking does not lead to plumage damage, but severe feather pecking results in feather loss [2]. Gentle feather pecking may develop into severe feather pecking. The risk of skin pecking is increased by severe feather pecking [3]. It is known that influencing factors besides a genetic predisposition, lies inter alia in housing, management and feeding [2,3,4,5]. A main causative factor that has been found in several studies is the inhibition of foraging behaviors in pullets and laying hens [5,6,7,8,9]. Light is necessary to enable normal behavior [10,11,12] and performance [12,13] in poultry. It can be assumed that the presence of natural daylight corresponds to the requirements of laying hens. Therefore, in Germany, laying hens kept in stables should be provided with daylight inlets in accordance with the local animal welfare legislation [14,15]. Old windowless barns, preventing any daylight from entering, have to be converted. However, in case a disproportionate effort is required due to technical or other reasons, windows can be substituted by appropriate artificial light sources [15]. A light spectrum in the range of 400 to 700 nm plus a proportion of 4–5% ultraviolet light (UV-A light) in the range of 320 to 400 nm are recommended in laying hen husbandry [16]. It is known that the retinae of birds are sensitive to ultraviolet radiation [17] as well as blue, green and red light [18,19]. Bennett and Cutthill [19] described three functions of UV sensitivity within birds: orientation, foraging and sexual selection. Furthermore, it is supposed that UV light might function in signaling between birds, for example, in individual recognition [20]. The lack of UV light may cause birds to perceive the plumage of other hens differently. Perhaps in the absence of UV light, plumage markings are not visible for conspecifics and might possibly promote feather and injurious pecking at conspecifics [20]. 

In general, this means that light spectrum influences both the behavior and performance of laying hens [12]. It was shown that laying hens [21], as well as turkeys [22], prefer fluorescent tubes to incandescent light and layers housed in blue light weighed less than those housed in fluorescent light [23]. Sherwin [22] postulated the preference for fluorescent light mimics daylight. Behavioral studies indicate that physical activities of hens might be greater in fluorescent lighting as opposed to incandescent lighting [24,25]. The effect of UV-light on the occurrence of feather pecking in poultry is a controversially discussed issue. Previous studies on turkeys showed that feather pecking and pecking injuries [26,27] were not affected by light sources. On the other hand, the provision of UV radiation in combination with environmental enrichment significantly reduced the incidence in culling because of pecking injuries of male turkeys [28]. Laying hens exposed to daylight including UV spectrum had reduced feather damage and cannibalism tendencies [29]. Prescott et al. [17] concluded that a lack of UV-light might encourage feather pecking in laying hens. It is also known that birds reared without access to natural light until adult age may have behavioral problems because of their inability to adapt to new housing environments [30,31]. Other studies found that UV-light provision during rearing increased the risk of abnormal behavior during laying [32,33].

UV light influences the birds’ perception, the reflectivity of their plumage and affects bird behavior. Nonetheless, little is known about the detailed effects of UV-A lighting during the entire rearing and production period on a practical indoor farm level. The aim of this on-farm study was to investigate the effects of UV-A light provided in addition to standard lighting on performance and the condition of the integument of pullets and laying hens. It was hypothesized that the provision of UV-A light to hens may reduce feather pecking and this may lead to improved plumage conditions and fewer skin injuries. 

## 2. Materials and Methods 

### 2.1. Research Ethics and Animals 

The birds were housed in accordance with EU [34] and national law [14,15]. Federal law included the German Animal Welfare Act (German designation: TierSchG) [14], national requirements for animal husbandry (German designation: TierSchNutztV) [15] and the Animal Protection Guideline for Laying Hens and Pullets with Intact Beaks of Lower Saxony, Germany (German designation: Niedersächsische Empfehlungen) [35]. In compliance with the European Directive 2010/63/EU [36], the experiment did not imply any invasive treatment of the hens. This study was reviewed by the Animal Welfare Officer of the University of Veterinary Medicine Hannover, Foundation (TVO- 02-04-2015) as well as by the Working Group “Laying Hens” of the Animal Welfare Programme of Lower Saxony, Germany. 

About 92,000 Lohmann brown classic hens (Lohmann Tierzucht GmbH, Cuxhaven, Germany) were included in the study on a commercial rearing and production farm in Germany. The hens with intact beaks were housed during rearing (May to September) and laying period (September to May) in a barn with an aviary system (Rearing: NATURA-Rearing, Fa Big Dutchman International AG, Vechta, Germany; Production: NATURA-nova twin Fa Big Dutchman International AG, Vechta, Germany). 

The pullets were housed as day-old chicken on a rearing farm in three identically constructed barns up to the 16th/17th week of life (WL). Each barn was divided into 15 pens housing about 1800 (parietal pens) and about 2550 (central pens) pullets. The chicks were randomly distributed to the pens. The stocking density of 18 pullets/m^2^ of usable floor space was in accordance with the Animal Protection Guideline for Laying Hens and Pullets with Intact Beak of Lower Saxony [35]. After rehousing, the hens were scientifically monitored until the 48th week of life on a commercial farm in two identical barns with ten pens of about 4600 hens each. During production, the stocking density was 18 hens/m^2^ of usable floor space, which is in accordance with the national requirements for animal husbandry [14].

All birds were managed under the same practical conditions in accordance with standard procedures with commercial feed, a commercial feeding regime and standard lighting program, access to manipulable material as well as usual litter management. Pullets and laying hens were fed with commercial meal-type feed. During the different growing and production phases, different feed variations were used. A three-phase feeding program (Chick Starter, Grower, Developer and Pre-layer) was performed for the pullets as well as for the layers (Layer Starter, Layer Phase 2 and 3). An increased content of crude fiber (5–6%) was used in all phases. In addition, both groups were offered grit twice a month in the scratching area. The lighting program started 10 days after the arrival of the chickens in the rearing farm with a lighting period of 14 h of light (L) and 10 h of darkness (D). The duration of lighting was reduced during growing to 8L:16D in the 7th week of life. In the laying farm, the lighting program started with 9L:14D at 18/19 weeks of life and was gradually extended until 14L:10D (week 25). The barns were littered with wood shavings and enriched with bales of alfalfa hay and pecking stones. In case of severe feather pecking or injurious pecking, finely chopped straw was supplied extensively into the scratching area twice a week. 

### 2.2. Light Spectrum and Intensity

During both rearing and production, half of the hens (about 460,000 hens) were housed under commercial light conditions without UV-A light (UVexcl group), while the other half was additionally exposed to UV-A light (UVincl group) in addition to standard lighting in the range of about 315–380 nm. 

Therefore, 20 of the 45 pens during rearing as well as ten of the 20 pens (about 46,000 hens) during production received an additional UV-A light to illuminate the whole barn. The barns during rearing were windowless. On the production farm, natural light with slight UV-radiation entered the stable through the windows located in the outdoor corridors at both barns. Therefore, the UV-range of the light spectrum only reached a very limited area in the barns. In order to minimize light shining straight into the litter area by sunspots, blinds were closed depending on the amount of solar radiation. As a consequence of the appearance of behavioral disorders during production, the windows were first closed incompletely, then completely closed on the sunny side and at the end on both sides of both barns. 

**Figure 1:**
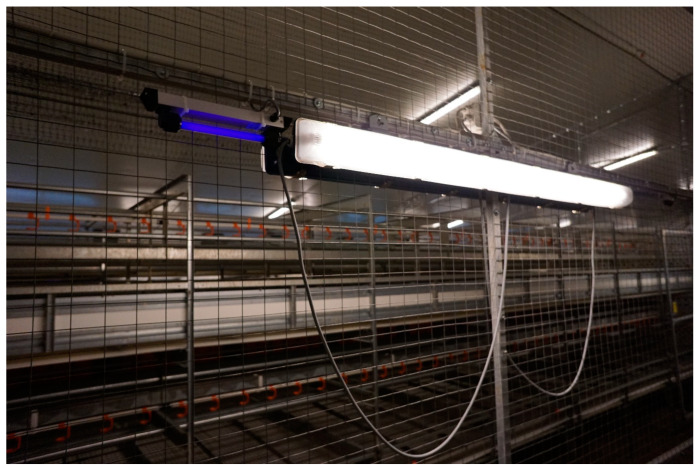
Standard fluorescent lamps (emitting warm white light) as well as black light tubes (emitting long-wave UV-A radiation) were additionally provided in the poultry barn in order to simulate the daylight spectrum in the groups with UV-A light (UVincl).

At the outset of the experiment, no light sources for poultry houses emitting the full daylight spectrum including UV-A light were commercially available. Therefore, standard fluorescent tubes (Rearing farm: warm white Osram Lumilux T8 L 58W/830, Osram GmbH, Munich, Germany; Production farm: warm white Omnilux 6W 230V G5T5, Osram GmbH, Munich, Germany) as well as black light tubes (Philips TL-D 36W BLB 1SL; Philips GmbH Market DACH, Hamburg, Germany) were additionally provided to emit long-wave UV-radiation simulating daylight (Figure 1). In the UVexcl groups, commercial lighting conditions were set up using the standard fluorescent tubes. In the rearing farm, we used five to six lamps per pen, placed at the side of the corridors (parietal pens) or at the top (central pens). In the production farm, nine to 12 lamps were installed per pen, located under the portal frame (three to four lamps), at the side of the corridors (three to four lamps) as well as at the top (three to four lamps). In the UVincl groups, the standard fluorescent tubes were installed in the same way as in the UVexcl group. Additional UV-lamps (Omnilux 6W 230V G5T5, Osram GmbH, Munich, Germany) were placed alongside the standard fluorescent tubes. In the rearing farm, five to six UV-lamps per pen were also placed on the side of the corridors (parietal pens) or on the top (central pens). In the production farm, UV lamps were installed under the portal frame (three to four lamps) and on the side of the corridors per pen (three to four lamps). 

All installed types of light sources were tested for flicker-free operation (GL Specis 1.0 touch, GL Optic/Just Normlicht GmbH, Weilheim/Teck, Germany). Standard fluorescent tubes and the black light tubes were controlled separately by the stable technology to the desired UV-A percentage in the total spectrum of the barn lighting of approx. 4–5%. Throughout the laying phase, the light intensity was gradually reduced in both barns as a consequence of higher plumage losses and skin injuries. At week 48, the UV-A tubes were turned off completely for the UVincl group, as no further dimming was possible. 

The spectral analysis was performed using a spectrometer (Model X4, Light Analyzer Gigahertz Optik GmbH, Türkenfeld, Germany). A luxmeter (TES-1336A, TES Electrical Electronic Corp, Taipei, Taiwan) was used to measure the light intensity (a three-point measuring system was used to create an average at each point in the barn). The measurements were taken at animal height in the scratching area and in the aviary system at three times points (4th WL, 8th WL, 14th WL) during rearing and monthly during the laying phase. Overall, measurements were carried out at five pens per lighting group in pullet rearing and at all 10 pens per lighting group in the laying phase. The spectral analysis was performed in the scratching area at one point per pen during rearing (resulting in a total of five measuring points per lighting group) and at two measuring points per pen in the production period (resulting in a total of 20 measuring points per lighting group). During rearing, the measurement of light intensity was carried out in five pens at one point in the scratching area and in the aviary at two points (resulting in a total of 15 points per lighting group). During laying, seven points per pen were taken to measure the light intensity (resulting in a total of 70 measuring points per lighting group) in the two lighting groups. Therefore, five points in the scratching area and two points in the aviary system were selected. For further analysis, mean and standard deviation per pen and lighting group were calculated monthly for the results of spectral analysis and light intensity.

### 2.3. Production Parameters 

Egg production was recorded daily for each barn (UVincl group and UVexcl group) from the 19th to 48th week of life. The laying rate (%) was presented as average number of eggs laid per week, referred to the number of hens housed at the beginning of the laying phase. Furthermore, the cumulative egg production per hen (eggs/hen housed) was calculated at the end of the experiment in week 48. 

Dead birds were collected daily by the farmer to count the weekly as well as the cumulative mortality rate (%) of each group. The weekly mortality rate was calculated based on the number of hens that died in the respective week of life in relation to the number of hens housed at the start of the experiment. To calculate the cumulative mortality, the weekly mortality rates were added. Moreover, the farmer checked all dead or killed birds for signs of cannibalism. Therefore, the occurrence of skin injuries with a minimum size of about 2 cm (yes/no) were documented for each dead hen. It was also recorded if a bird died due to smothering (dead hens were found accumulated on a pile, often in a corner or on the wall of a pen). 

### 2.4. Plumage Damage, Skin Injuries and Body Weight 

The hens were visited weekly, starting at the third week of life. As the UV-A lamps were turned off due to the appearance of behavioral disorders in the 48th week of life, data are shown until then. During the weekly visits, a total of 200 randomly selected birds per group (10 birds per pen during rearing and 20 birds per pen during production) were individually weighed, feather scored and examined for injuries of the skin. The hens were randomly selected from all parts of the barn as well as all parts of the aviary system.

The hens were weighed with a poultry scale (Manual Poultry Scale FlexScale, Big Dutchman, International AG., Vechta, Germany) first at week 17 up to week 48. At the end of rearing, the uniformity of the flock weight was calculated. The uniformity is the percentage of individuals within 10% of the mean body weight of the hens. The plumage and the skin were visually scored by two trained observers for damage weekly, using a scoring system of 0 to 3 with higher scores indicating more severe damage (Table 1). This scoring system was adapted and modified from the method described by Tauson et al. (2005) [37], being in accordance with Lower Saxony’s Animal Protection Guideline for Laying Hens and Pullets with Intact Beaks [35]. Therefore, the bird’s body was divided into five regions, including the neck, back, wings, tail, and belly (including cloaca). For each body region, a score was defined for assessing plumage damage as well as for measuring skin injuries. To calculate the interobserver reliability for feather damage and skin injuries, 52 randomly selected birds were used. These plumage scores were cumulated to a whole body score per hen with a maximum of 15 points (worst case). For further analysis of skin injuries, the worst injury score per hen was taken. 

Additional management strategies were taken at first signs of feather damage or cannibalism following a graduated emergency scheme in accordance with Lower Saxony’s Animal Protection Guideline for Laying Hens and Pullets with Intact Beaks (for details see [35]). The emergency scheme was started if, per visit (independent of body region), at least 25% of the scored hens per lighting group showed extensive feather damage and feather loss (score ≥2) or if injuries were detected in ≥10% of the scored hens per lighting group. 

### 2.5. Data Presentation and Statistical Analysis 

Statistical analysis was conducted using SPSS Statistics (version 26; IBM Corp., Armonk, NY, USA). Data for each pen of the two lighting groups (Group 1: UVincl; Group 2: UVexcl) were treated as a statistical unit. Due to the large overall data set, feather scores were summarized to a whole feather score per hen. For skin injuries, the worst injury score per hen was taken for further analysis and data presentation, the following target variables were calculated: whole plumage score per hen, proportion of hens with skin injuries on at least one body region, cumulative mortality and body weight. Inferential statistics were calculated to test for the effect of lighting group on plumage loss, skin injuries, body weight and mortality in the studied pens. Egg production data were averaged for all weeks (18th to 48th WL) and analyzed only descriptively. Initially, data were tested for normality using a normal plot including Gaussian distribution curve and a Shapiro–Wilk test. The Levene procedure was used to test for homoscedasticity. To obtain normality, data were log transformed (plumage damage and skin injuries). Data were analyzed using generalized linear models (two-way analysis of variance) to test the effects of UV lighting and pen on dependent variable body weight, plumage damage, skin injuries and mortality. Fixed factors were light group and pen. The week of life within light group was added as a covariate. For all dependent variables, the analyses were done across the week and for each week separately. All post hoc pairwise comparisons of the pens were adjusted according to Bonferroni correction. Differences between the tested parameters were found significant if p-values were *p* < 0.05 and the confidence intervals were 95.0%. 

The interobserver reliability was calculated using the Krippendorff’s alpha with the ‘macro’ developed by Hayes and Krippendorff [38]. Each body region was calculated separately, which resulted in values for the interobserver reliability of the neck, back, wings, tail, and belly. 

## 3. Results

### 3.1. Housing Parameters

**Figure 2:**
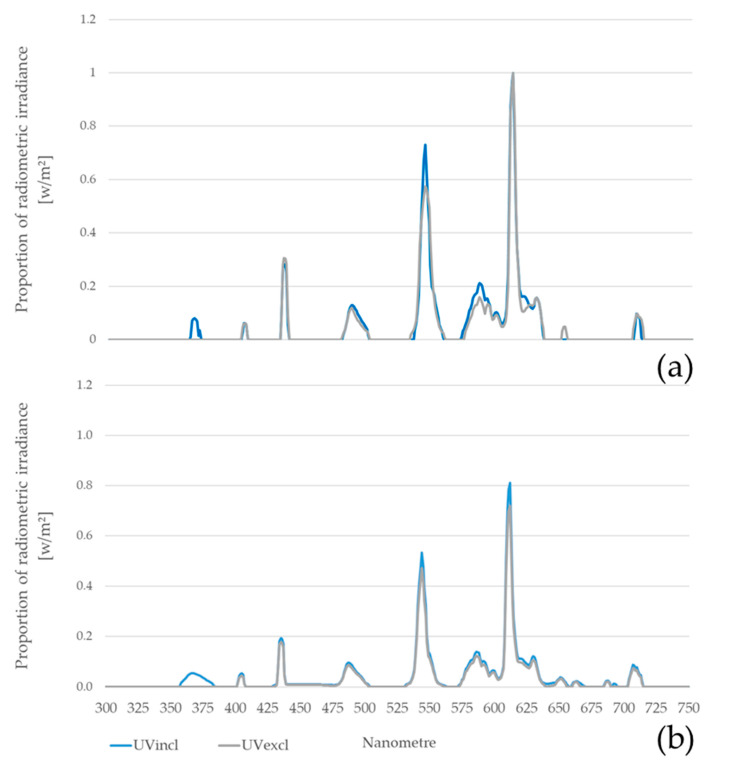
Light spectrum during rearing (a) and laying (b) with (UVincl) and without (UVexcl) additional UV-A light with peaks in the wavelength in yellow (about 525–550 nm) and red (about 600–625 nm). A peak of ultraviolet light (about 350–380 nm) was only measured in the UVincl group.

The measured light spectrum during rearing and laying without and with additional UV-A light is shown in Figure 2. A peak of ultraviolet light (about 357–380 nm) was only detected in the barn with additionally installed UV-lamps with approx. 4–5% of the total spectrum. 

**Figure 3:**
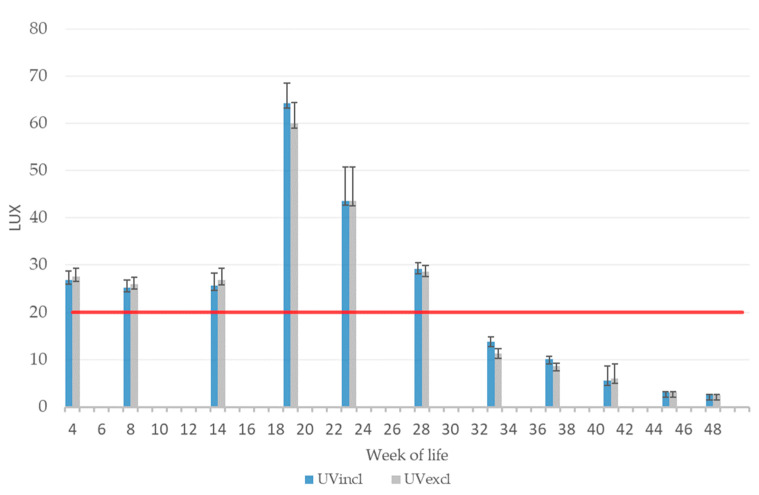
Mean light intensity (lux) during rearing and production with (UVincl) and without (UVexcl) additional UV-A light (red line: European recommendation of the minimum light intensity in laying hen houses).

During rearing, the measured mean light intensity (lux) was 25.3 ± 1.51 lux to 26.9 ± 2.33 lux in the UVincl group and 25.9 ± 1.51 lux to 27.6 ± 1.84 lux in the UVexcl group, therefore meeting the European recommended minimum light intensity of 20 lux (Figure 3). At the start of laying (18th/19th week of life), the average light intensity in the two lighting groups was 64.0 ± 6.60 lux (UVincl group) and 60.0 ± 4.38 lux (UVexcl group), respectively. In general, during production, the light intensity was continually reduced in both light groups to 2.41 ± 0.14 lux (UVincl group) and 2.4 ± 0.13 lux (UVexcl group) at the end of this study (last reading at week 48). The reduction in the light intensity in both barns was necessary because of feather pecking and cannibalism in both groups and was carried out in consultation with the veterinary surgeon. This was a part of the action plan in case of severe feather pecking and, therefore, still in line with EU legislation. 

### 3.2. Production Data

Table 2 shows production parameters during rearing and laying in relation to the light spectrum. In comparison with the UV-A light treatment, the group without UV-A light showed higher mortality rates during production (F(1,9) = 19, *p* = 0.45) and significantly more dead hens with injuries were detected (F(1,9) = 14.21, *p* < 0.05). There was an increase in losses in both groups immediately after rehousing from the 20th week of life onwards. During this time period up to the laying peak in week 27, smothering was observed, resulting in a cumulative mortality rate of birds which had died due to smothering of 1.24% (UVincl group) and 1.15% (UVexcl group) (F(1,9) = 1.94, *p* = 0.16). 

**Figure 4:**
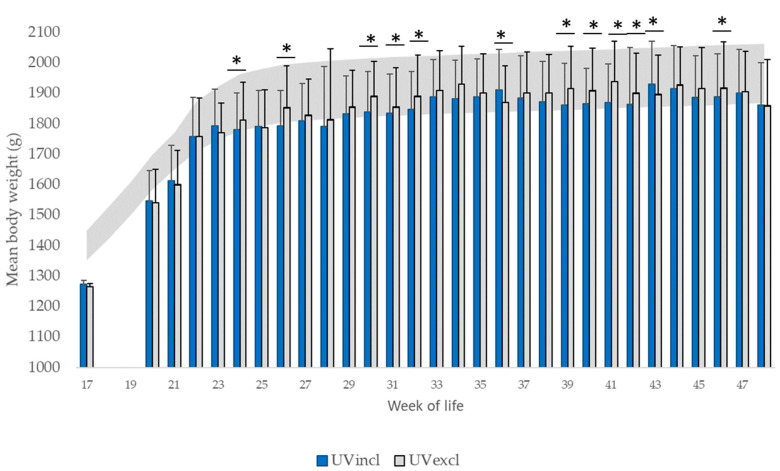
Mean body weight (g) at the end of rearing (17th WL) and during production (18th–48th week of life) in hens (*n* = 200 hens/lighting group and week of life) as well as breeder’s recommendation of target minimum and maximum value for production (grey area). * Significant differences in the average live weight (*p* < 0.05; WL: 24, 26, 30, 31, 32, 36, 39, 40, 41, 42, 43, 46).

The average body weight at the end of rearing as well as at the end of the experiment ranged with only slight deviations between the two lighting groups (F(15,304) = 1.94, *p* = 0.19). Nevertheless, light spectrum had a significant effect on the average live weight (F(15,7663) = 10.11, *p* < 0.05) during laying at several visits, where the mean body weights of the hens housed with standard lighting were heavier compared to the hens housed with additional UV-light (Figure 4). During laying, the measured average body weights of both lighting groups were usually at the minimum value for production or even below the recommended value in the Breeder´s Management Guide for Alternative Systems [39]. With the onset of laying at week 21 (both groups) up to week 48, the recorded egg production was comparable. Throughout the entire rearing period and up to the end of the observation period (48th WL) during production, the health condition of the flocks was good, therefore medical treatment was not necessary.

### 3.3. Plumage Loss and Skin Injuries

The calculation of the interobserver reliability for feather damage resulted in a Krippendorf’s Alpha between 0.41 (wing) and 0.74 (back) and a value of 0.73 for injuries in all scored body regions.

**Figure 5:**
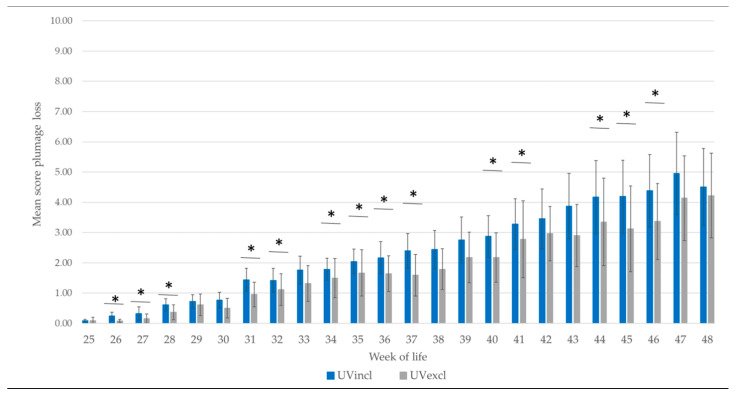
Mean plumage score of the whole body in hens (*n* = 200 hens/lighting group and week; Score 0 = intact plumage) during production period dependent on light spectrum (UV-A light +/−). The group with additional UV-A (UVincl) had a higher mean plumage score compared to the group without UV-A lighting (UVexcl). * Significant differences in the mean Plumage Score (*p* < 0.05; WL: 26, 27, 28, 31, 32, 34, 35, 36, 37, 40, 41, 44, 45, 46).

During rearing in both lighting groups, the plumage condition was still intact and no pullet with skin injuries was observed. The plumage loss of the scored hens during production is provided as an average of the sum of the scored five body regions (neck, back, wings, tail, and belly) in Figure 5. The plumage condition of the hens was intact in both groups at the start of laying (18th–24th WL) and declined with age. Plumage loss started in week 25. From the 27th week of life, a significantly higher mean plumage score (= poorer condition of the plumage) was found in the group with additional UV-A light compared to the group without (F(1,9) = 307.08, *p* < 0.05). At the end of the observation period (48th WL), the mean plumage score was above the mean score of four in both lighting groups, indicating more severe damage to the plumage compared to the beginning. The emergency scheme was started, if more hens with extensive feather loss were observed. In this case, new additional manipulable material—finely chopped straw—was placed into the litter in the scratching area two times a week starting in week 36 (UVincl group) and week 38 (UVexcl group) to calm down the situation. Due to plumage loss and skin injuries in both lighting groups, a gradual reduction in light intensity was also necessary (Figure 3). 

**Figure 6:**
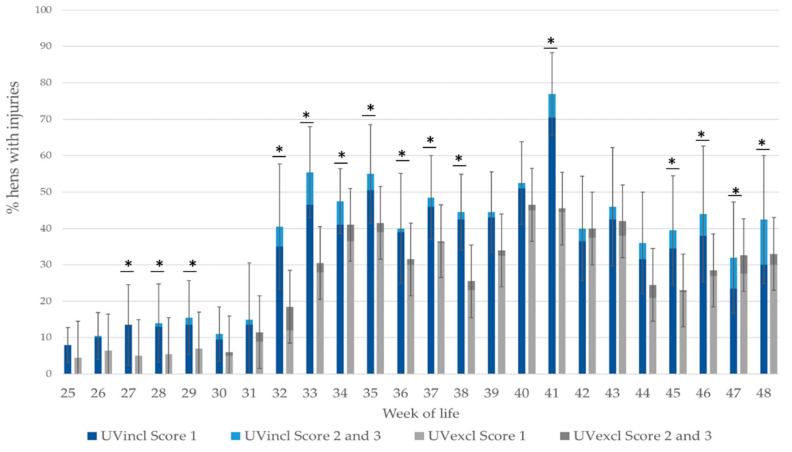
Percentage of hens with skin injuries on at least one body region (*n* = 200 hens/lighting group and week) during production period depending on light spectrum (UV-A light +/−). The UVincl group (blue bars) contained significantly more hens with severe injuries compared to the experimental group with standard lighting (UVexcl group; grey bars). * Significant differences in the percentage of hens with skin injuries (score 1 or higher) on at least one body region (*p* < 0.05; WL: 27, 28, 29, 32, 33, 34, 35, 36, 37, 38, 41, 45, 46, 47, 48).

A higher percentage of hens with severe injuries (Score 2 or 3) was found in the laying period in the group which was housed with additional UV-A light compared to the group with standard lighting (F(1,9) = 101.53, *p* < 0.05; Figure 6). During production, hens with severe injuries were detected in week 26 for the first time. A strong increase in the number of injuries was observed subsequently in the UVincl group with additional UV-A light in the 29th WL. At this time, 2.0% of the assessed hens were affected by skin injuries. In contrast, in the UVexcl group without UV-A light, no hens were affected (F(1,9) = 7.698, *p* < 0.006). Further peaks were registered about four weeks later (33th WL, F(1,9) = 19.01, *p* < 0.05) as well as in the 41th WL (F(1,9) = 10.45, *p* < 0.05) and 48th WL (F(1,9) = 12.84, *p* < 0.05). Up to week 48, the light intensity was reduced gradually down to 2.4 lux in both lighting groups. From this point on, no further dimming of the UV lamps was possible. Therefore, these had to be turned off in the UVincl group and thus, no further data analysis was carried out. 

In case of skin injuries, the emergency scheme approves the application of salt or magnesium as a countermeasure. Therefore, salt was added to the birds´ drinking water in both groups for five days starting at week 40 in addition to the above mentioned measures to prevent feather loss. In weeks 43 and 47, magnesium (Emgevet^®^, Verla-Pharm Arzneimittel GmbH & Co. KG, Tutzing, Germany) was administered via the drinking water to calm the situation down, too. 

## 4. Discussion

The present study determined under practical conditions whether the housing factors of up to 5% UV-A light in the total spectrum affected the occurrence of plumage and skin injuries during the rearing and production period of laying hens. Furthermore, production-related aspects were considered. It is known that birds‘ eyes are ultra-violet sensitive [16]; this is probably contributing to hue perception [19]. The lack of UV light may lead to abnormal plumage perception of conspecifics [20]. Thereby, the presence of natural “daylight” including UV radiation closely mimics their requirements. 

In general, it is known that feather damage and skin injuries occur during rearing and production as a consequence of feather pecking and cannibalism [40]. Bličik and Keeling [40] found a significant correlation between measured feather-pecking behavior and the extent of plumage damage. On-farm studies showed that non-beak-trimmed flocks were affected in more than 60% of cases, with plumage damage at the end of laying [40,41,42,43,44,45]. Results of the present study indicated plumage damage already at the onset of laying, with severely affected hens at the end of the experiment in both non-beak-trimmed groups. Nevertheless, a higher incidence of hens with plumage damage and skin injuries were present in the group with additional UV-A light compared to standard lighting. There may be a number of reasons for this finding. One possible explanation is that the offered UV-A light may have led to hens perceiving the light shining brighter than the standard lighting. In order to take into account the light intensity perceived by the bird’s eye, which differs from human perception, it is recommended to calculate the light intensity in Gallilux. An exemplary calculation using the results of the spectral analysis of both lighting groups (10 measurements per lighting group once a month) during production shows that it was up to 2.1% brighter in the UVincl group compared with the UVexcl group before the reduction of light intensity was carried out because of feather pecking and skin injuries. However, as soon as the light intensity was reduced starting in week 33, these differences in brightness of light could no longer be measured. Nevertheless, some of the results may be explained by brightness differences promoting the hens´ behavior in the following. On the other hand, it is known that the activity of hens increases with fluorescent lighting [42], and it is possible that pecking behavior may also increase. In order to answer this question, behavioral observation would be necessary when providing additional UV-A light to hens. 

The higher percentage of affected hens with plumage damage and skin injuries housed with additional UV-A light compared to standard illumination is possibly related to the type of illumination supply, too. At the outset of the current study, no light sources for poultry houses emitting the full daylight spectrum were commercially available. Therefore, in this study, the UV-A light was offered separately in addition to the standard lighting. The UV-A lamps were located directly near the standard illumination to create a “daylight” spectrum. It is possible that this type of installation caused a “separation” of the light spectrum including the UV-A light. However, this hypothesis was not detectable by the spectrometer. Recent studies have also shown that under natural conditions in the bush-rich habitat of the jungle hen, the UV radiation may be lower than applied in this farm study (4–5% of the total spectrum) [16]. This may possibly have led to a disturbed perception of the husbandry system and which may have resulted in abnormal reflectivity of the hens´ plumage and, consequently, in an increased occurrence of feather pecking. On the other hand, as already the pullets in the UVincl group were reared with a light spectrum including 4–5% UV-A illumination, they may have been adapted during laying to the appearance their conspecifics and their environment under prevailing lighting conditions. Depending on the results of the current field study, the technical implementation as well as the amount of UV-A radiation in poultry lighting seem to be important parameters when implementing daylight spectrum in commercial layer husbandry.

In addition to the influence of the light quality examined in this field study, other factors such as genetic aspects and nutritional imbalances, additional housing aspects as well as management factors during the entire life cycle trigger abnormal behavior [2,4]. These influencing factors could not be fully standardized as the current study was conducted on farm under practical field conditions. It is known that the occurrence of earlier feather damage during rearing [40,41,43] is a risk factor for feather damage later on in life. Even if there was no evidence of plumage damage and skin injuries, it is still possible that the rearing period has contributed to the occurrence of plumage damage during laying. It is known that feather losses in rearing are difficult to identify because lost feathers may be rapidly replaced in pullets [2,3]. However, the high frequency of farm visits (weekly) makes this seem rather unlikely. Although no feather damage occurred in all groups during rearing, first obvious plumage damage was seen shortly after rehousing in week 25. The affected plumage condition at an early age is in line with Drake et al. [33]. Nevertheless, it is also commonly thought that body weights below the breeder’s recommended target weight [37] and lacking uniformity of flock weight are risk factors for feather pecking and cannibalism, too. For 17-week-old Lohmann brown pullets, the breeder recommends [39] a mean live weight of 1400 g (range of 1351–1449 g). The uniformity is classified as “good” between 80% and 85% and as “medium” between 70% and 80%. At the end of rearing (17th WL), the mean weights of the pullets of both tested lighting groups were 9.10% (UVincl group) and 9.64% (UVexcl group) lower than this recommendation. The uniformity was 79.00% (UVincl group) and 81.50% (UVexcl group) and therefore met the breeders´ recommendation. The documented mean body weight below that recommendation of the Breeder‘s Management Guide in the tested groups, at the age when the hens are still growing and start laying, is a potential risk factor for early feather damage due to feather pecking [44]. The reduced weight development during rearing, the probably stressful transport to the production farm as well as the onset of laying are possibly associated with the decline in plumage condition in both lighting groups during the early laying period. 

In general, the occurrence of severe feather damage and skin injuries in both lighting groups caused by an outbreak of cannibalism made it necessary to take countermeasures to control the situation. Therefore, various measures as for example offering additional foraging material, dimming the light intensity as well as administering salt and magnesium via the drinking water, were performed in both groups. Even if the housing conditions were standardized as far as possible, nonetheless, they maybe have influenced the current investigations. For example, it is possible that the supply of additional manipulable material (finely chopped straw) at different times in the two light groups influenced the occurrence of feather pecking differently. Dimming the lighting was carried out in both barns at the same time in consultation with the veterinary surgeon. It is known that reducing light intensity decreases birds’ activity as well as aggressive behavior and cannibalism [43]. Kjaer and Vestergaard [45] showed that severe feather pecking was less frequent with three than with 30 lux. Even though the light intensity was extremely reduced in our study to about 2 lux in both groups, apparently cannibalism could be reduced, but not completely stopped. However, at week 48, the light reduction also meant that the UV-A tubes in the UVincl group were turned off. 

In the present study, an effect of the illumination including UV-A light was shown on production parameters during laying such as cumulative mortality and the number of recorded dead birds with injuries. On the other hand, the average weights of pullets at the end of rearing and on the production farm as well as laying performance were not affected by the spectrum of light offered. In the UVincl group lower mortality rates as well as fewer dead hens with pecking injuries were found compared to the UVexcl group. Conversely, in the current study, the plumage and skin condition were higher in groups with supplemented UV-A light during production. This discrepancy in the occurrence of increased skin and feather damage and the reduced mortality rate (in total and the number of dead birds with injuries) in the UVincl group could have had different causes. One possible cause is that feather pecking and injurious pecking are more common in UV-A light, but this damage to the animal does not necessarily lead to an increased mortality rate. Rather, the increased losses in the groups without UV-A light may be explainable due to illness. The barn without UV-A light experienced a problem with a bacterial infection with E. coli, starting after the 50th week of life (not shown). Conceivably, the animals of the UVexcl group already had a subclinical infection, which led to increased animal losses. In order to answer this question, pathological observations would be necessary. 

## 5. Conclusions

In conclusion, this on-farm study showed no clear beneficial effects from using supplemental 4–5% UV-A light in the total spectrum of barn lighting with regards to improved plumage conditions or reduced skin injuries. In our study, additional UV-A light did not prevent plumage damage and skin injuries in layers as plumage condition was worse in hens housed with additional UV-A light. It appears that, therefore, additional management and housing factors as well as nutrition play a role in reducing or preventing behavioral disorders. Further studies should include light tubes containing the bird’s optimum percentage of UV-A light in the total light spectrum, as well as behavioral observations. Moreover, further studies are needed to find out more about the appropriate level of UV supplementation, most suitable light sources, besides the optimum light spectrum for laying hens. 

## Figures and Tables

**Table 1:** Scoring system of plumage and skin condition of the hens in accordance with the Animal Protection Guideline for Laying Hens and Pullets with Intact Beak of Lower Saxony [35].

Score	Plumage Damage	Skin Injuries
0	Intact plumage	No skin injuries
1	Moderate feather damage and feather loss	<1.0 cm
2	Extensive feather damage and feather loss patterns	>1.0–2.0 cm
3	Mostly featherless area; completely denuded	>2.0 cm

**Table 2:** Production data (mean ± SD) during rearing and production in experimental group without UV-A light (UVexcl) and with additional UV-A light (UVincl).

Parameter	UVincl Group	UVexcl Group
**Mortality (%) Cumulative**		
Rearing period (0–17th WL)	1.82 ± 0.07 ^a^	1.48 ± 0.11 ^a^
Production period (18th–48th WL)	4.54 ± 1.20 ^a^	5.27 ± 1.36 ^b^
**Mortality (%) Cumulative with Injuries**		
Rearing period (0–17th WL)	0.00	0.00
Production period (18th–48th WL)	0.87 ± 0.14 ^a^	1.30 ± 0.36 ^b^
**Mortality (%) Cumulative Caused by Smothering**		
Rearing period (0–17th WL)	0.00	0.00
Production period (18th–48th WL) *^1^	1.24 ± 0.77 ^a^	1.15 ± 0.31 ^a^
**Live Weight (g)**		
End of rearing (17th WL) (mean)	1,272.60 ± 105.89 ^a^	1265.00 ± 97.75 ^a^
min–max	1261–1289	1245–1288
Uniformity (%)	79.00 ± 2.88 ^a^	81.50 ± 6.53 ^a^
End of experiment (48th WL) (mean)	1862.16 ± 152.13 ^a^	1859.40 ± 138.91 ^a^
min–max	1534.00–2406.00	1532.00–2247.00
Egg Production *^2^		
eggs/hen housed (18th–48th WL)	158.32	162.15
Mean laying rate/ housed hen (48th WL)	82.60 ± 1.13	81.63 ± 0.97

^a/b^ Means within a row differ significantly (*p* ≤ 0.05). *^1^ Smothering were observed from 18th–27th week of life. *^2^ Egg production was analyzed only with descriptive statistics.
